# Knowledge and Attitude of Undergraduate Health Professions Students towards Hepatitis B and C

**DOI:** 10.1155/2023/6699940

**Published:** 2023-09-30

**Authors:** Ali Rashash R. Alzahrani

**Affiliations:** Mathematical Science Department, College of Applied Sciences, Umm Al-Qura University, 17 Altaef Road, Makkah 21955, Saudi Arabia

## Abstract

The current study sought to establish the attitude and knowledge level of medical science students in Tibah University towards hepatitis B and C. A cross-sectional study involving 369 students drawn from the faculties of medicine, dentistry, applied medical sciences, pharmacy, nursing, and medical rehabilitation sciences was conducted where a standardised questionnaire was used to determine the attitude and knowledge level of undergraduate students drawn from the college of medicine in Tibah University, Saudi Arabia, regarding viral infections. With a mean of 0.71, 0.69, and 0.66 and a standard deviation (SD) of 0.24, 0.34, and 0.24 for virology and transmission, symptoms and clinical outcomes, and treatment and prevention, respectively, the knowledge level of the health professions students towards hepatitis B and C infections was significant. The knowledge level about the infections was higher among male students than among female students. Similarly, students in their final years of medical school had significantly higher knowledge levels about hepatitis B and C than their counterparts in their first years of medical school. There was also a positive correlation between the attitude of the health professions students towards the disease and their knowledge levels of the disease. Findings indicate that enhanced instruction on the nature, virology, transmission, symptoms, treatment, clinical outcomes, and prevention of hepatitis B and C can help improve the knowledge levels and attitude of the health professions students towards the disease and its management.

## 1. Background

Hepatitis is a viral infection that causes inflammation and/or liver damage, thus impeding its ability to fight infections, filter blood, and process nutrients. The onset of cases of this inflammatory condition of the liver is attributed to hepatitis viruses, but it can also be triggered by toxins including alcohol and some medications or by certain medical conditions among them autoimmune diseases [1]. Although there are 5 main hepatitis viruses, namely, hepatitis A, B, C, D, and E, the hepatitis B virus (HBV) and hepatitis C virus (HCV) comprise the most common types of infectious viruses that cause the liver disease [1, 2]. Hepatitis B is a serious infection of the liver triggered by exposure to the hepatitis B virus which can be easily averted through vaccination [3, 4]. Hepatitis C is a liver disease triggered by infection of the liver by the hepatitis C virus causing it to be inflamed [5].

Hepatitis B and C constitute a great health concern owing to the related burden of illness and death the infections herald and the likelihood of epidemic spread and outbreaks they portend. Viral infections are the biggest causes of liver disease and damage and have the potential to progress to liver cancer, cirrhosis, and chronic liver disease which are both debilitating and difficult and costly to manage [5, 6]. Each year, hepatitis B and C viruses are responsible for approximately 600,000 and 350,000 deaths, respectively, across the world [6]. Globally, there are about 240 million carriers of chronic HBV, of whom, about 0.25 percent die annually [4], while annual HCV infections stand at approximately 4 million people [5], highlighting the prevalence and gravity of the disease.

Viral hepatitis constitutes a significant public health concern in the Middle Eastern region. With a prevalence of over 3.5% for HCV [7] and an HBV prevalence rate of between 2 and 8% [8], the region has a high incidence of the infections and lower intermediate to high endemicity. Saudi Arabia, in particular, has an HBV prevalence rate of 3.02%, indicating a lower intermediate endemicity of viral hepatitis [8]; this is attributable to the high rate of hepatitis B vaccination among its population which has helped lower the rate of infections. The related mortality and morbidity have not recorded a similar trajectory highlighting the seriousness of viral hepatitis as a public health issue in the country and the role nonadherence to therapy, low diagnosis rates, and poor disease awareness play in amplifying the burden of the disease among Saudis [9].

Percutaneous injuries constitute one of the most common modes of transmission of blood-borne pathogens such as HBV and HCV and hence the susceptibility of health personnel and health professions students to the infections [2]. Given the centrality of adequate knowledge and the right attitude towards hepatitis infections in preventing the transmission of the infections, healthcare workers and health professions students can play a critical role in the prevention of infection by promoting knowledge about the disease among the patients and the population at large [9]. Health professions students on the path to becoming health professionals are at risk of contracting HBV and HCV through percutaneous injuries and hence the centrality of their general attitude and knowledge about HBV and HCV in keeping the infections in check. The fact that both HBV and HCV are associated with diverse extrahepatic manifestations, affect the liver and could lead to hepatocellular carcinoma and cirrhosis, and can be transmitted in the same way through exposure to body fluids and blood from unsafe health care and unsafe injection practices [10, 11] highlights the need to study the two diseases together.

The current study sought to establish the attitude and knowledge level of health professions students in Tibah University towards HBV and HCV diseases.

## 2. Materials and Methods

### 2.1. Study Population

The study population comprised health professions students pursuing medicine, dentistry, applied medical sciences, pharmacy, nursing, and medical rehabilitation sciences, in the college of medicine of Tibah University, Saudi Arabia. The training and background of the students in the different professions in basic sciences and in particular virology and clinical management of patients with hepatitis B and C vary across the various years of study. Junior health professions students in the first and second year have a significantly lower general understanding and knowledge of HBV and HCV compared with their senior counterparts in the fourth and fifth year [12]. Research has shown that while the junior students tend to have an awareness of the viral infection, they lack adequate knowledge about its nature, risk factors, and causative agent, a trend significantly associated with their academic levels [12]. Thus, compared to senior health professions students, junior health professions students have inadequate awareness and lack knowledge regarding HBV and HCV, their risk factors, transmission mediums, and prevention strategies.

### 2.2. Sample

The study sample comprised 369 undergraduate students drawn from six faculties, namely, medicine, dentistry, applied medical sciences, pharmacy, nursing, and medical rehabilitation sciences, in the college of medicine of Tibah University, Saudi Arabia. A cross-sectional survey based on the sample was conducted in early 2022 to establish the knowledge levels and attitudes of the health professions students with regard to HCV and HBV infections.

Calculation of the sample was affected on the basis of the random subgroup's positive knowledge proportion as well as the consideration of a type I error of 0.05 and a precision of 0.06. An invitation of the possible participants for the study was done using leaflets and posters that were distributed across the college of medicine, which requested interested students to participate in the study. Those who expressed interest were provided with the information sheet which clearly explained what the survey was about before proceeding to sign the informed consent forms.

### 2.3. Questionnaire

A standardised questionnaire, derived from and developed based on the questionnaires used in the surveys by Joukar et al. [13], Mansour-Ghanaei et al. [2], and Richmond et al. [14], was employed to collect data about the attitudes and knowledge levels of the health professions students in relation to hepatitis B and C. The questionnaire comprised four sections: the first section comprised 12 questions that sought to collect the demographic data of the students participating in the study; the second section comprised 28 questions relating to the students' knowledge levels about HBV; the third section comprised 29 questions testing the knowledge levels of the students about HCV; and the fourth section comprised 18 questions testing the attitude of the students towards patients with HBV and HCV.

Cronbach's alpha coefficient was employed to confirm the reliability and validity of the questionnaires. For questions testing HCV knowledge, alpha was equal to 0.9. For questions testing HBV knowledge, alpha was equal to 0.8. With a Cronbach's alpha coefficient of greater than 0.7, the internal reliability and validity of the HBV and HCV knowledge testers were good. For HCV and HBV attitude questions, alpha was equal to 0.67, indicating a not so good internal reliability and validity and hence the potential of the attitude testers not being reliable measures of the variable. A correlation test conducted also established that there was no multicollinearity between the questionnaires and hence the potential for high reliability of the statistical inferences of this study.

Each demographic group was employed to establish the mean knowledge score. Four statements were used to establish the attitude of the students towards HCV and HBV by indicating whether the statements were “True” or “False.”

Demographic data of the students participating in the study including their gender, institution, academic year, faculty (medicine, dentistry, applied medical sciences, pharmacy, nursing, and medical rehabilitation sciences), history of training or working in hospital or clinical practice, and family history of hepatitis were collected.

The questionnaires were administered through one-on-one interviews conducted and filled by qualified medical professionals among the team of researchers. Informed consent forms were signed prior to the commencement of the interviews.

### 2.4. Statistical Analysis

Data collected were analysed using the IBM SPSS 29.0.0.0 software. Descriptive statistics analysis and in particular frequency, standard deviation (SD), and mean, as well as comparison means including one-way analysis of variance (ANOVA), were performed. Statistical significance was pegged at *P* <0.05.

## 3. Results

A total of 369 health professions students participated in the cross-sectional survey, among whom, 194 were female and 175 were male. In terms of academic year, 37 of the students were first year, 106 second year, 62 third year, 98 fourth year, and 66 fifth year, and more. In terms of faculty, 49 of the students were studying medicine, 18 dentistry, 134 applied medical sciences, 42 pharmacy, 77 nursing, and 49 medical rehabilitation sciences. Among the students, only 128 (34.7%) had a history of training or working in hospital or clinical practice, while the remaining 241 (65.3%) did not have such an experience. While only 32 (8.7%) students had a family history of hepatitis, 337 (91.3%) of them did not have a family history of hepatitis.

Knowledge levels towards HBV and HCV were analysed through 10 questions. The mean (SD) of the knowledge level regarding HBV and HCV was 0.68 ± 0.437 based on the 10 questions relating to virology and transmission, symptoms and clinical outcomes, and treatment and prevention of the infections.  Table 1 illustrates the proportion of correct answers regarding HBV and HCV knowledge questions.

Attitudes of the health professions students towards HBV and HCV infections were assessed using 4 questions. The responses of the students towards the statements gauging their attitudes are presented in Table 2. The mean (SD) of the attitude of the students was 0.71 ± 0.449 with the maximum mean score being 0.81 and a minimum 0.58.

One-way ANOVA was performed to compare the knowledge level among students regarding virology and transmission, symptoms and clinical outcomes, and treatment and prevention of the HBV and HCV infections. It was established that there was no statistically significant difference in the knowledge levels of the students regarding the viral infections with 8 of the 10 knowledge questions having a *P* value of greater than 0.05, as illustrated in Table 3. However, there was a statistically significant difference in the knowledge levels of the health professions students on whether the viruses can be transmitted through contaminated food and water and whether persons infected with the viruses develop clear symptoms of the infections given that the two knowledge questions had *P* values of less than 0.005, that is, *P* = 0.33 and *P* = 0.002, respectively.

There was also no statistically significant difference in the attitudes of the students towards HBV and HCV. As shown in Table 3, in 3 out of the 4 indicative elements, *P* values obtained were greater than 0.05, that is, *P* = 0.090, *P* = 0.998, and *P* = 0.637. However, there was a statistically significant difference in the attitudes of the students in relation to their willingness to provide care to people infected with hepatitis B or C viruses, an attitude statement that had a *P* value of 0.43.

A one-way ANOVA was also conducted to compare the effect of the various demographic characteristics on the knowledge levels and attitudes of the health professions students. The analysis established that there was no statistically significant difference in the knowledge levels and attitudes of the students towards HBV and HCV based on their gender (*P* = 0.374), faculty (*P* = 0.318), history of training or working in hospital or clinical practice (*P* = 0.397), and family history of hepatitis (*P* = 0.235). There was however a statistically significant difference in the knowledge levels and attitudes of the students towards HBV and HCV based on their academic year (*P* = 0.045) (Table 4).

There was also a high frequency of correct answers compared to wrong answers, as illustrated in Table 5. The mean frequencies for correct answers compared to wrong answers for the questions testing the knowledge levels of the students were 252.6 or 68.5% and 116.4 or 31.5%, respectively.

Concerning attitude questions, as provided in Table 6, the frequency of “True” responses was higher for each of the four statements compared to that of the “False” responses. The average frequencies of affirmative and negative responses to the questions were 259.5 (70.3%) and 109.5 (29.7%), respectively.

## 4. Discussion

The students have a moderate level of knowledge towards virology, transmission, symptoms, clinical outcomes, treatment, and prevention of HBV and HCV infections, as indicated by the 68.5% correct responses to the 10 knowledge level statements (Table 5). This affirms the findings of an earlier study on the attitude and knowledge of health workers towards HBV conducted by Ahmadi et al. [15] and later surveys by Razi et al. [16] and Ahmad et al. [17] which showed that medical personnel had moderate knowledge levels about hepatitis B and C, thus highlighting the need to strengthen the knowledge levels of the healthcare workers and health professions students towards HBV and HCV.

The current study also found no significant difference (*P* = 0.375) in the knowledge levels about HBV and HCV among female and male health professions students who participated in the study (Table 1). This contrasts with the findings of an earlier survey by Ahmadi et al. [15] which found female medical workers to be more knowledgeable about the viral infections than their male counterparts and the recent study by Ahmad et al. [17] which showed a significant correlation between the gender of medical workers and their levels of knowledge regarding the viral infections.

The results also showed that most of the correct answers concerned virology and transmission (260.5 or 70.5%), followed by symptoms and clinical outcomes (at 255.5 or 69.2%), and then treatment and prevention (at 243.5 or 66%) (Table 5). This sharply contrasts with the findings of the study by Ahmadi et al. [15] which showed the knowledge of medical personnel concerning the nature of the disease to be lowest as well as that of a similar survey by Ghahramani et al. [18] which found students' knowledge about the prevention of the viral infections to be highest compared to all other aspects.

The results of the survey also showed no significant difference in knowledge levels among the students based on their history of hepatitis, faculty, history of training, and clinical experience. This could be because all the survey participants were health science students. However, there was a variation in knowledge levels about HBV and HCV among students in different academic years with knowledge levels increasing with the years of study effectively affirming the recent findings by Ahmad et al. [17] that the knowledge and skills students have about the viral infections improve as they advance in their study.

The survey also showed that the participating students had a more positive attitude towards HBV and HCV infections. This is indicated by the virtue of 215 (58.3%) of the students being comfortable shaking hands with infected persons, the willingness of 298 (80.8%) of the students to provide care to infected persons, the enthusiasm of 272 (73.7) of the students to provide the same standard of care they offer other patients to people with HBV and HCV, and the preference of 253 (68.6%) of the students to wear double gloves when treating infected persons. The findings are consistent with those of earlier surveys by Ahmad et al. [17] and Mansour-Ghanaei et al. [2] in which a majority of participants had a more positive attitude towards handling, provision of care, and interaction with HBV and HCV patients.

The results further demonstrate a more significant association between the knowledge level of the participating students and their attitude towards HBV and HCV such that attitude towards the disease is more positive with the increasing knowledge about the infections. This could be attributed to the relatively better knowledge and understanding of the disease by a majority of the students. Similar studies conducted in different countries showed attitude towards HBV and HCV is commensurate with the knowledge levels of students about the infections; the higher their knowledge levels are, the more positive their attitudes towards the disease [17, 19, 20]. This is however inconsistent with the recent survey by Quang et al. [21] which showed that knowledge does not change the attitude of health professions students towards the disease.

Nonetheless, the relatively small sample size and cross-sectional design of this study contribute to the limitations and weaknesses of the data collected and analysed. The present study is also centered on the participants who positively responded to the call to participate in the survey while ignoring those who declined the invitation. A major strength of the study is its focus on a pressing public health concern and a population susceptible to disease and with the potential to significantly impact its management. Enhanced awareness of HBV and HCV is critical for students who are essentially future healthcare practitioners.

## 5. Conclusion

Knowledge and attitude of students pursuing health science in the Middle East are poor to moderate particularly among students with minimal interaction with infected persons. A significant positive correlation was established between the knowledge levels of health professions students and their attitude towards the disease.

Adequate knowledge and proper attitude towards the infections affect the readiness and enthusiasm of medical personnel to interact with and provide care to the infected people. Enhanced education on virology, transmission, symptoms, clinical outcomes, treatment, and prevention can go a long way in improving the readiness of the medical staff to treat and manage HBV and HCV as well as interact with and handle the infected. This will not only help reduce the high occupational risk the infections pose to medical staff but also minimise discriminatory practices towards hepatitis B and C patients by medical workers.

## Figures and Tables

**Table 1:** Correct knowledge answers about HBV and HCV infections.

Statements	Correct answers Female, *N*(%)	Correct answers Male, *N* (%)	*P* value
(1) The causative agent of hepatitis B and C is a virus	168 (86.6)	145 (82.9)	0.319 (NS)
(2) Hepatitis B and C can be transmitted through contaminated food and water	108 (55.7)	78 (44.6)	0.033
(3) Hepatitis B and C can be contracted through needle stick or sharp object injury	163 (84.0)	157 (89.8)	0.108 (NS)
(4) Kissing can transmit hepatitis B and C	115 (59.3)	107 (61.1)	0.716 (NS)
(5) All individuals infected with hepatitis B or C develop clear symptoms of hepatitis	130 (67.0)	89 (50.9)	0.002
(6) Hepatitis B and C can cause liver cirrhosis and cancer	152 (78.4)	140 (80)	0.698 (NS)
(7) There is no pharmaceutical treatment for hepatitis B and C	107 (55.2)	108 (61.7)	0.203 (NS)
(8) Proper disposal of needle and sharps is a way of hepatitis B and C prevention	166 (85.6)	147 (84)	0.676 (NS)
(9) HBV can be prevented by vaccination	143 (73.7)	139 (79.4)	0.197 (NS)
(10) HCV can be prevented by vaccination	85 (43.8)	79 (45.1)	0.798 (NS)
Averages	133.7 (68.9)	118.9 (67.9)	0.375 (NS)

^*∗*^NS indicates no significance.

**Table 2:** Positive attitude answers about HBV and HCV infections.

Statements	True statement Female, *N* (%)	True statement Male, *N* (%)	*P* value
(1) I feel comfortable to shake hands of a person infected with hepatitis B and C	105 (54.1)	110 (62.9)	0.090 (NS)
(2) I am willing to provide care to people with hepatitis B or C	149 (76.8)	149 (85.1)	0.043
(3) When treating hepatitis B and C patients, I will provide the same standard of care as I do for other patients	145 (74.7)	127 (72.6)	0.637 (NS)
(4) When treating hepatitis B and C patients, I would prefer to wear double pairs of gloves	133 (68.6)	120 (68.6)	0.998 (NS)

^*∗*^NS indicates no significance.

**Table 3:** Knowledge and attitudes of the health professions students in relation to virology and transmission, symptoms and clinical outcomes, and treatment and prevention aspects of HBV and HCV (ANOVA).

Question/statement		Sum of squares	df	Mean square	F	Sig.
The causative agent of hepatitis B and C is a virus	Between groups	0.129	1	0.129	0.997	0.319 (NS)
	Within groups	47.373	367	0.129		
	Total	47.501	368			

Hepatitis B and C can be transmitted through contaminated food and water	Between groups	1.133	1	1.133	4.565	0.033
	Within groups	91.111	367	0.248		
	Total	92.244	368			

Hepatitis B and C can be contracted through needle-stick or sharp object injury	Between groups	0.298	1	0.298	2.594	0.108 (NS)
	Within groups	42.195	367	0.115		
	Total	42.493	368			

Kissing can transmit hepatitis B and C	Between groups	0.032	1	0.032	0.133	0.716 (NS)
	Within groups	88.407	367	0.241		
	Total	88.439	368			

All individuals infected with hepatitis B or C develop clear symptoms of hepatitis	Between groups	2.401	1	2.401	10.171	0.002
	Within groups	86.624	367	0.236		
	Total	89.024	368			

Hepatitis B and C can cause liver cirrhosis and cancer	Between groups	0.025	1	0.025	0.151	0.698 (NS)
	Within groups	60.907	367	0.166		
	Total	60.932	368			

There is no pharmaceutical treatment for hepatitis B and C	Between groups	0.396	1	0.396	1.626	0.203 (NS)
	Within groups	89.333	367	0.243		
	Total	89.729	368			

Proper disposal of needle and sharps is a way of hepatitis B and C prevention	Between groups	0.023	1	0.023	0.175	0.676 (NS)
	Within groups	47.479	367	0.129		
	Total	47.501	368			

HBV can be prevented by vaccination	Between groups	0.301	1	0.301	1.668	0.197 (NS)
	Within groups	66.187	367	0.180		
	Total	66.488	368			

HCV can be prevented by vaccination	Between groups	0.016	1	0.016	0.065	0.798 (NS)
	Within groups	91.095	367	0.248		
	Total	91.111	368			

I feel comfortable to shake hands of a person infected with hepatitis B and C virus	Between groups	0.702	1	0.702	2.893	0.090 (NS)
	Within groups	89.027	367	0.243		
	Total	89.729	368			

I am willing to provide care to people with hepatitis B or C	Between groups	0.640	1	0.640	4.141	0.043
	Within groups	56.699	367	0.154		
	Total	57.339	368			

When treating hepatitis B and C patients, I will provide the same standard of care as I do for other patients	Between groups	0.043	1	0.043	0.223	0.637 (NS)
	Within groups	71.458	367	0.195		
	Total	71.501	368			

When treating hepatitis B and C patients, I would prefer to wear double pairs of gloves	Between groups	0.000	1	0.000	0.000	0.998 (NS)
	Within groups	79.534	367	0.217		
	Total	79.534	368			

**Table 4:** Knowledge and attitudes of the health professions students in relation to the demographic characteristics (ANOVA).

Variables		Sum of squares	df	Mean square	F	Sig.
Gender	Between groups	1.064	4	0.266	1.065	0.374 (NS)
	Within groups	90.941	364	0.250		
	Total	92.005	368			

University	Between groups	0.000	4	0.000		
	Within groups	0.000	364	0.000		
	Total	0.000	368			

Academic year	Between groups	16.053	4	4.013	2.463	0.045
	Within groups	593.172	364	1.630		
	Total	609.225	368			

Faculty	Between groups	11.108	4	2.777	1.183	0.318 (NS)
	Within groups	854.247	364	2.347		
	Total	865.355	368			

History of training or working in hospital or clinical practice	Between groups	0.926	4	0.231	1.019	0.397 (NS)
	Within groups	82.673	364	0.227		
	Total	83.599	368			

Family history of hepatitis	Between groups	0.442	4	0.110	1.396	0.235 (NS)
	Within groups	28.783	364	0.079		
	Total	29.225	368			

**Table 5:** Responses to knowledge questions.

Knowledge questions	Correct answers, *N* (%)	Wrong answers, *N* (%)	Missing values
Virology and transmission			
(1) The causative agent of hepatitis B and C is a virus	313 (84.8)	56 (15.2)	0
(2) Hepatitis B and C can be transmitted through contaminated food and water	186 (50.4)	183 (49.6)	0
(3) Hepatitis B and C can be contracted through needle stick or sharp object injury	320 (86.7)	49 (13.3)	0
(4) Kissing can transmit hepatitis B and C	222 (60.2)	147 (39.8)	0
Symptoms and clinical outcomes			
(5) All individuals infected with hepatitis B or C develop clear symptoms of hepatitis	219 (59.3)	150 (40.7)	0
(6) Hepatitis B and C can cause liver cirrhosis and cancer	292 (79.1)	77 (20.9)	0
Treatment and prevention			
(7) There is no pharmaceutical treatment for hepatitis B and C	215 (58.3)	154 (41.7)	0
(8) Proper disposal of needle and sharps is a way of hepatitis B and C prevention	313 (84.8)	56 (15.2)	0
(9) HBV can be prevented by vaccination	282 (76.4)	87 (23.6)	0
(10) HCV can be prevented by vaccination	164 (44.4)	205 (55.6)	0
Average frequencies	252.6 (68.5)	116.4 (31.5)	0

**Table 6:** Responses to attitude questions.

Attitude questions	True response, *N* (%)	False response, *N* (%)	Missing values
(1) I feel comfortable to shake hands of a person infected with hepatitis B and C virus	215 (58.3)	154 (41.7)	0
(2) I am willing to provide care to people with hepatitis B or C	298 (80.8)	71 (19.2)	0
(3) When treating hepatitis B and C patients, I will provide the same standard of care as I do for other patients	272 (73.7)	97 (26.3)	0
(4) When treating hepatitis B and C patients, I would prefer to wear double pairs of gloves	253 (68.6)	116 (31.4)	0
Averages	259.5 (70.3)	109.5 (29.7)	0

## Data Availability

The datasets used and/or analysed during the current study are available from the corresponding author upon reasonable request.
